# Shell Thickness Dependence of Interparticle Energy Transfer in Core-Shell ZnSe/ZnSe Quantum Dots Doping with Europium

**DOI:** 10.1186/s11671-018-2541-2

**Published:** 2018-04-23

**Authors:** Ni Liu, Shuxin Li, Caifeng Wang, Jie Li

**Affiliations:** 1 College of Aeronautical Engineering, Binzhou University, Shandong, 256603 China; 2 Anhui Key Laboratory of Nanomaterials, and Technology and Key Laboratory of Materials Physics, Institure of Solid State Physics, Chinese Academy of Sciences, Hefei, 230031 China

**Keywords:** Core-shell quantum dots, Energy transfer, Shell thickness, Fluorescence lifetime

## Abstract

Low-toxic core-shell ZnSe:Eu/ZnS quantum dots (QDs) were prepared through two steps in water solution: nucleation doping and epitaxial shell grown. The structural and morphological characteristics of ZnSe/ZnS:Eu QDs with different shell thickness were explored by transmission electron microscopy (TEM) and X-ray diffraction (XRD) results. The characteristic photoluminescence (PL) intensity of Eu ions was enhanced whereas that of band-edge luminescence and defect-related luminescence of ZnSe QDs was decreased with increasing shell thickness. The transformation of PL intensity revealed an efficient energy transfer process between ZnSe and Eu. The PL intensity ratio of Eu ions (*I*_613_) to ZnSe QDs (*I*_*B*_) under different shell thickness was systemically analyzed by PL spectra and time–resolved PL spectra. The obtained results were in agreement with the theory analysis results by the kinetic theory of energy transfer, revealing that energy was transmitted in the form of dipole-electric dipole interaction. This particular method of adjusting luminous via changing the shell thickness can provide valuable insights towards the fundamental understanding and application of QDs in the field of optoelectronics.

## Background

Rare earth (RE) doped chalcogenide semiconductor quantum dots have received particular attention in the field of nanomaterials, due to their excellent photoelectric properties, such as multispectral luminescence, long fluorescent life, high luminous efficiency, low-gentle magnetic, etc. [1–4]. However, the absorption cross section of RE ions is very small (the order of magnitude is 10^− 21^ cm^− 2^), which leads to low luminescence efficiency [5]. Moreover, it is very difficult to directly stimulate transition of RE ions, since the f-f transition belongs to the parity forbidden transition according to the selection rule [6]. In order to overcome the above mentioned restrictions, significant research efforts have been devoted to the doping of RE ions into luminescent matrix materials. The matrix materials with large absorption cross-section can transfer energy to RE ions, so as to indirectly enhance their luminescence. This phenomenon is known as the “antenna effect” [7]. Various materials, such as fluorides, silicates, and chalcogenide semiconductor quantum dots are usually employed as matrix materials [8–14]. Among these, chalcogenide semiconductor quantum dots have some unique properties, such as quantum size effect, high fluorescence efficiency, large absorption cross section (1.1 × 10^− 18^ cm^− 2^), light stability, rendering them as excellent candidate materials [15–18]. Up to now, the research efforts on RE doping in chalcogenide semiconductor quantum dots were mainly focused on tuning luminescence wavelength and improving PL efficiency, by adjusting doping concentration, reaction time, and other experimental parameters [19–21]. In the research of dopant QDs, energy transfer was usually a means of explaining spectral phenomena, but the intrinsic mechanism of energy transfer was rarely explained.

In view of the above perspectives, the PL characteristics and intrinsic energy transfer mechanism of core-shell ZnSe:Eu/ZnS QDs were thoroughly explored in the present work. The luminescence spectra of the ZnSe host materials and Eu ions were investigated by controlling shell thickness. The mechanism of energy transfer between Eu ions and ZnSe/ZnS core-shell quantum dots was systematically analyzed by time-resolved fluorescence spectroscopy and energy transfer kinetic theory.

## Methods/Experimental

In this paper, ZnSe:Eu/ZnS core-shell quantum dots were prepared through nucleation doping and epitaxial growth method. The detailed preparation process was described as follows: the mixture of zinc nitrate hexahydrate(Zn (NO_3_)_2_.6H_2_O), europium(III) nitrate hexahydrate(Eu (NO_3_)_3_.6H_2_O), and 3-Mercaptopropionic acid(MPA) with a molar ratio of Zn^2+^/Eu/MPA = 1: 0.06: 20 prepared under stirring in N_2_ atmosphere. Then 50 mL of 0.5 M sodium selenohydride (NaHSe) solution was injected into the precursor solution of Zn rapidly followed by condensation at 100 °C under continuous stirring. Afterwards, ZnSe:Eu nanoparticles were purified by employing absolute ethanol and centrifugal precipitation. For obtaining ZnS shell by epitaxial growth method, 20 mg of ZnSe: Eu nanoparticles were added to 100 mL of deionized water and were stirred in N_2_ atmosphere until obtaining a clear and transparent solution. Then, zinc acetate (Zn(AC)_2_.2H_2_O, 0.1 M)) and MPA (0.7 mL) with a pH of 10.3 were added dropwise to the ZnSe: Eu solution and were heated at 90 °C in N_2_ atmosphere until the reaction completed. The same absolute ethanol and centrifugal precipitation purification process was used. Pure ZnSe:Eu/ZnS QDs were obtained which put into a vacuum oven for further use. The samples used for characterization were all re-dissolved in deionized water.

The size and morphology of ZnSe:Eu/ZnS QDs QDs were investigated by transmission electron microscopy (TEM) using Technai G2 operated at 200 kV. The XRD of the sample powder was performed by wide angle X-ray scattering with graphite monochromatized high intensity 0.148 nm Cu–Kα radiation. PL spectra were measured at room temperature using Jobin Yvon Fluorolog-3 system (Jobin Yvon Division Company, France) and excitation wavelength was 365 nm. The luminescence lifetime spectra of samples were measured relative to FLS920 fluorescence spectrophotometer equipped with a 450 W xenon lamp as the excitation source, and the pulse frequency is 100 ns.

## Results and Discussion

**Fig. 1:**
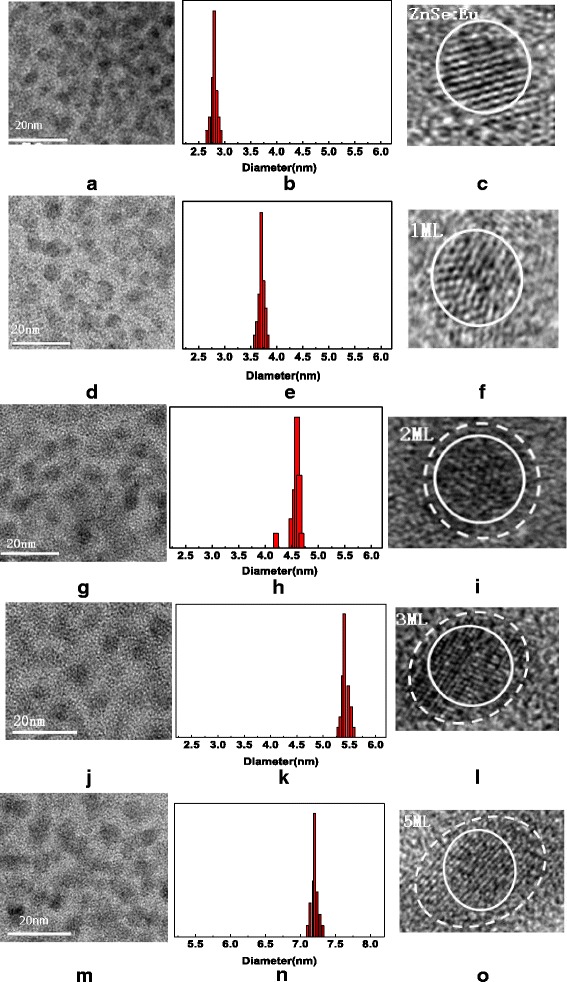
TEM images and histograms of the measured particle sizes of ZnSe:Eu QDs (**a**, **b**) and overcoated with 1 ML (**d**, **e**), 2 ML(**g**, **h**), 3 ML (**j**, **k**), and 5 ML (**m**, **n**) of the ZnS shell, respectively. Cryo-HRTEM of core ZnSe:Eu (**c**) images and the corresponding core-shell ZnSe:Eu /ZnS QDs with 1 ML (**f**), 2 ML (**i**), 3 ML (**l**), 5 ML (**o**) shell, respectively

Figure 1a–o representatively shows the TEM results for core ZnSe:Eu QDs and core-shell ZnSe:Eu/ZnS QDs with different shell thickness. From the Fig. 1a–c, we can see that the shape of ZnSe:Eu QDs are regular spherical, and the average size is 2.7 nm. The high-resolution transmission electron microscopy (HRTEM) demonstrates the excellent crystallinity of the ZnSe:Eu QDs. When ZnS shell is epitaxially grown on the surface of ZnSe:Eu QDs, the size of the ODs became significantly larger, i.e., 3.6 nm (1 ML), 4.6 nm (2 ML), 5.4 nm (3 ML), and 7.2 nm (5 ML). As the thickness of the shell increases, the shape of the quantum dots gradually becomes ellipsoid, but the significant changing of lattice fringes in crystal boundaries between ZnSe and ZnS was not obvious due to the method of epitaxial growth.

**Fig. 2:**
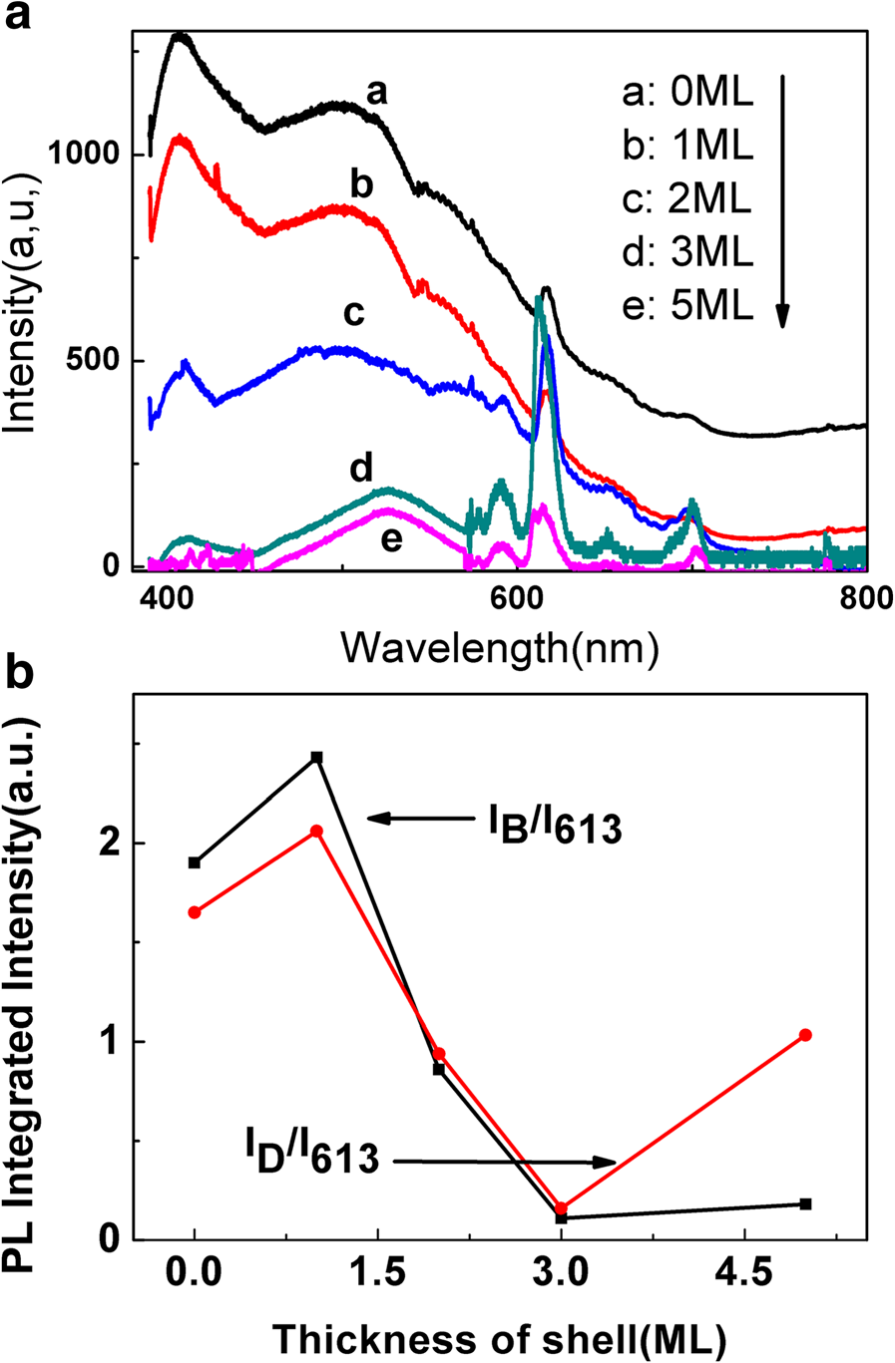
**a** PL spectra of core-shell ZnSe:Eu/ZnS QDs with different shell thicknesses. **b** Comparison of PL intensity ratio of Eu (*I*_613_) to the band edge (*I*_*B*_) of the ZnSe quantum dot as well as the defect-related (*I*_*D*_)

In order to further improve the fluorescence efficiency of the ZnSe:Eu QDs, the epitaxial shell growth of ZnS on the core of ZnSe:Eu is prepared. The PL spectra of core-shell ZnSe:Eu/ZnS QDs with different shell thicknesses is depicted in Fig. 2a. Three characteristic luminescence peaks of Eu are shown, which are ascribe to ^5^D_0_ → ^7^F_1_(590 nm), ^5^D_0_ → ^7^F_2_(613 nm), and ^5^D_0_ → ^7^F_3_(652 nm) [22], correspondingly. On the other hand, another two luminescence peaks of ZnSe QDs appeared, which are band-edge luminescence (406 nm) with a relatively sharp full width at half maximum (FWHM) and defect state luminescence (510 nm) with broad FWHM [23–25]. With the increase of ZnS shell thickness, the characteristic luminescence intensity of Eu is enhanced. When the thickness of the shell is 3 ML, the three characteristic luminescence intensities of Eu ions reach the maximum value, while the two PL intensities of ZnSe QDs are reduced, as shown in Fig. 2b. The PL intensity transformation of ZnSe:Eu QDs indicates energy transfer between ZnSe and Eu. The ratio of PL intensity integral of the Eu ion (*I*_613_) to the band edge PL intensity integral (*I*_*B*_) of the ZnSe quantum dot as well as the defect-related luminescence intensity (*I*_*D*_) were calculated, respectively. The results revealed that the energy transfer efficiency varies with the thickness of the shell layer.

**Fig. 3:**
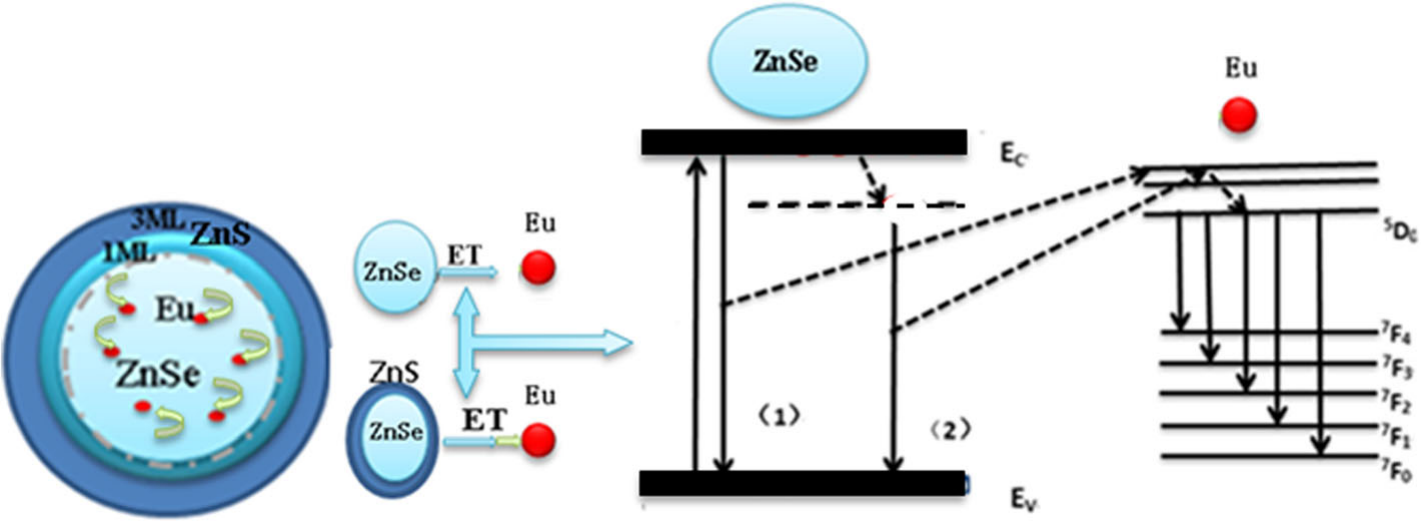
Proposed energy transfer mechanism between ZnSe (donor) and Eu (acceptor) in ZnSe:Eu/ZnS QDs. (1) Band-edge-related radiation recombination process. (2) Defect-state related radiation recombination process

In particular, when ZnSe:Eu QDs are epitaxial coated with ZnS shell, the lattice constants of the two counterparts are not equal and the lattice continuity across the interface is destroyed, resulting in lattice mismatch. Because of lattice mismatch, ZnSe suffered compressive stress at the interface and ZnS is subjected to tensile stress, and the average lattice constant changed [26]. Consequently, the induced stress modifies the energy level structure of the core-shell nanoparticles, which in turn alters the electron energy level structure in the nanocrystalline particles. Three possible steps are considered for exciton recombination process: (i) radiation recombination of excitons in host materials (including the edge emission and defect emission of ZnSe QDs); (ii) non- radiation recombination through heat transfer loss; (iii) energy transfer between ZnSe host and Eu ions, which enhanced PL intensity of Eu ions. These three steps competed each other, resulted in the simultaneously appearance of three PL peaks as shown in Fig. 2a. The two types of fluorescence transfer part of energy to the adjacent Eu ions during radiation recombination process, which resulted in electrons transitions in Eu ions from ^7^F_0_ state to ^5^D_0_ state [27], as shown in Fig. 3.

**Fig. 4:**
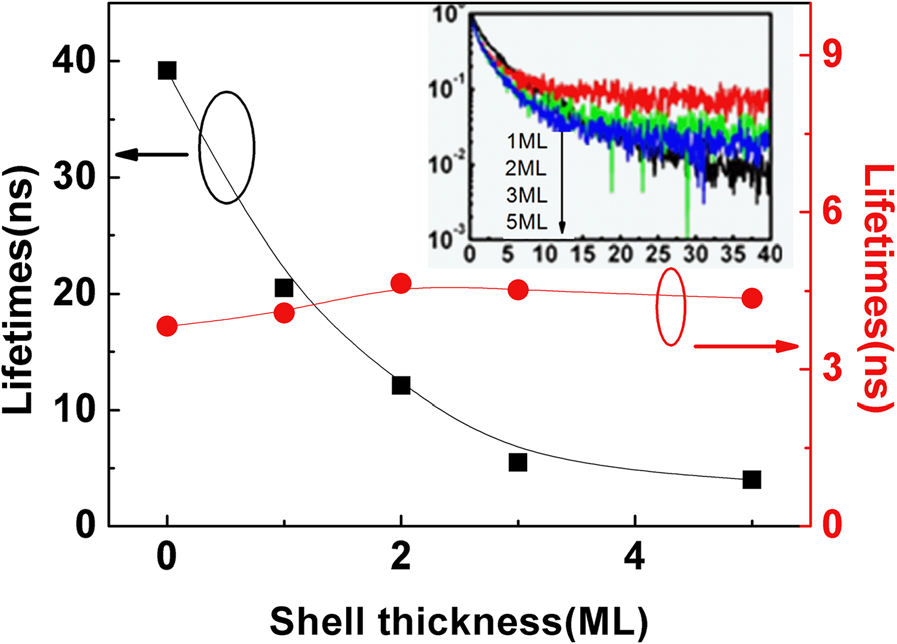
Fluorescence lifetime of ZnSe QDs (*I*_*B*_) and that of Eu (*I*_613_) with different Zne shell thickness. The inset is time-resolved PL spectra of band-edge luminescence peak of ZnSe QDs (*I*_*B*_) with different ZnS shell thickness

The time-resolved PL spectra of ZnSe:Eu/ZnS core-shell QDs is an important means to detect energy transfer between them [28]. The fluorescence lifetime of the characteristic luminescence peak at 613 nm of Eu and that of the band-edge luminescence peak at 406 nm of ZnSe with different ZnS shell thickness is shown in Fig. 4. With the increase of ZnS shell thickness, the average lifetime of donor ZnSe QDs decreases exponentially as fast-acting energy transfer for enhanced stress in core-shell structure. Concomitantly, the acceptor Eu average lifetime increases as it receives transferred photon energy.

According to the kinetic theory of energy transfer, the ratio of ZnSe band edge PL intensity (*I*_*B*_) to that of Eu ion (*I*_*E*_) as a function of the ZnS shell thickness can be calculated by time-resolved PL spectra [29]. Under steady-state excitation conditions, the energy transfer rate for ZnSe-Eu can be expressed according to Eq. 1:

1 $$ {W}_{\mathrm{ZnSe}-\mathrm{Eu}}{n}_1=\frac{n_2}{\tau_2} $$

where *W*_ZnSe − Eu_ is the energy transfer rate of ZnSe-Eu; *τ*_2_ is the lifetime of Eu ions (*I*_613_); *n*_1_ and *n*_2_ are the number of excited ions of ZnSe and Eu ion level, respectively. The macroscopic energy transfer rate can be expressed as follows:

2 $$ {W}_{\mathrm{ZnSe}-\mathrm{Eu}}=\frac{1}{\tau_1}-\frac{1}{\tau_0} $$

where *τ*_0_ is the lifetime of the bare ZnSe QDs when the ZnS shell thickness is 0 ML and *τ*_1_ is the lifetime of ZnSe band edges (*I*_*B*_). The ratio between band-edge emission intensity (*I*_*B*_) of ZnSe QDs to that of Eu ions (*I*_613_) can be expressed as follows:

3 $$ \frac{\gamma_2{\tau}_2}{\gamma_1}{W}_{\mathrm{ZnSe}-\mathrm{Eu}}=\frac{I_{613}}{I_B} $$

where *γ*_1_ and *γ*_2_ are the emissive coefficients.

**Fig. 5:**
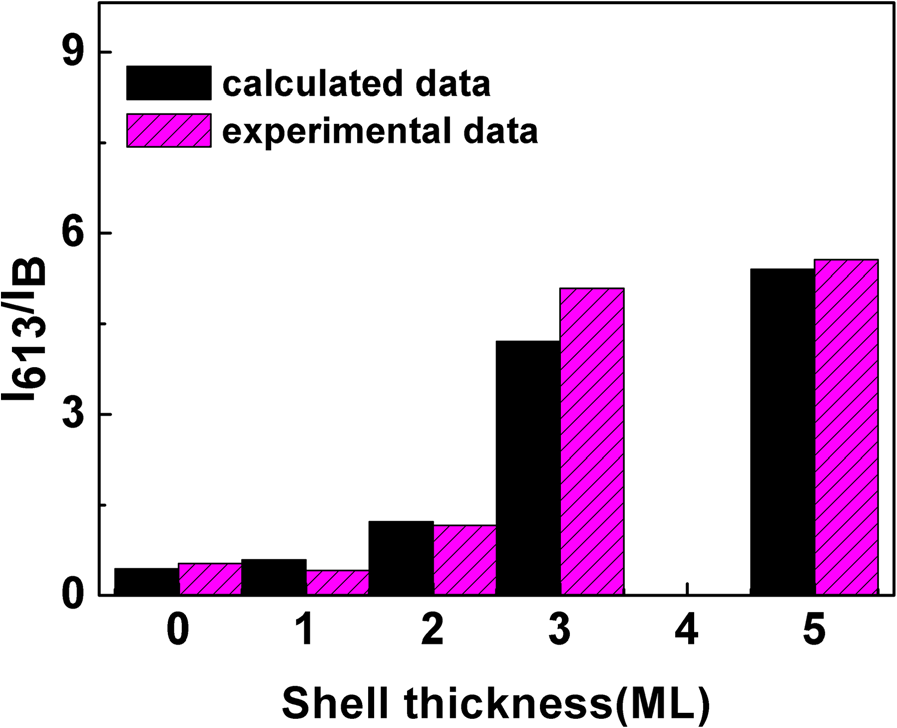
Comparison of theoretical and experimental values of *I*_613_/*I*_*B*_ of ZnSe:Eu/ZnS core-shell quantum dots with different shell thickness

Comparing the experimental ratio of *I*_613_/*I*_*B*_ (red bar graph) with the theoretical results (black bar graph), we can conclude that the ratio calculated by the luminescence kinetics model agree well with the experimental results, as shown in Fig. 5. It also demonstrates the energy transfer efficiency increased with the increase of shell thickness.

**Fig. 6:**
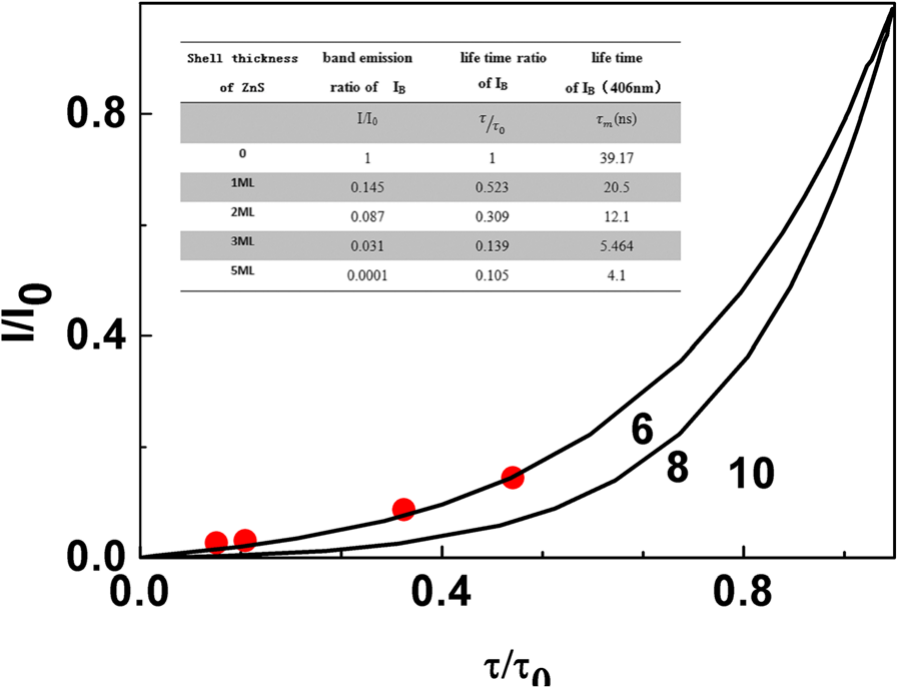
Fitting diagram of experimental and theoretical values of $$ \raisebox{1ex}{$I$}\!\left/ \!\raisebox{-1ex}{${I}_0$}\right. $$and $$ \raisebox{1ex}{$\uptau $}\!\left/ \!\raisebox{-1ex}{${\tau}_0$}\right. $$. The inset is PL ratio of ZnSe:Eu QDs to ZnSe:Eu/ZnS QDs and fluorescence lifetime ratio of them with different shell thickness

No radiation energy transfer mainly takes place via the interaction between multipolar moments. When the distance between the host and the guest is relatively short, the energy can be transferred from the host (donor: ZnSe) to the guest (acceptor: Eu) through multipole interaction [30]. The mechanism of energy transfer between donor and acceptor can be corroborated by considering the fluorescence intensity and lifetime of the donor and the acceptor. The fluorescence lifetime of the multipole moment can be expressed according to Eq. (4):

4 $$ \upvarphi \left(\mathrm{t}\right)=\exp \left[\frac{-t}{\tau_0}-T\left(1-\frac{3}{s}\right)\frac{c}{c_0}{\left(\frac{t}{\tau_0}\right)}^{\frac{3}{s}}\right] $$

where *τ*_0_ is the fluorescence lifetime of the donor without dopant, c is the doping concentration of acceptor, *c*_0_ is the critical concentration related to critical distance($$ {c}_0=\raisebox{1ex}{$3$}\!\left/ \!\raisebox{-1ex}{$4\pi {R}_0^3$}\right. $$)。Different S values stand for the interaction of different multipolar moments [31]. It corresponds to electric dipole-electric dipole interaction for *s* = 6, dipole-quadrupole interaction for *s* = 8, and quadrupole-quadrupole interaction for *s* = 10, respectively. The fitting results for different s values are depicted in Fig. 6. The ratio of band-edge luminescence intensity and fluorescence lifetime is well matched with the fitting results for *s* = 6, which indicates the existence of energy transfer between the donor of ZnSe and Eu acceptor by electric dipole-electric dipole mode. These two of the interactions for cross relaxation are electrostatic in origin.

## Conclusions

The ZnSe:Eu/ZnS (QDs) were prepared by wet chemical method via nuclear doping followed by epitaxial ZnS shell growth. The morphology and structure of core-shell ZnSe:Eu/ZnS QDs were clearly revealed by TEM and XRD results. The photoluminescence (PL) spectra of ZnSe:Eu/ZnS QDs with different thickness of ZnS shell showed that the PL intensity of the Eu characteristic luminescence peak increased while that of characteristic luminescence and defect luminescence of ZnSe decreased, illustrating an effective energy transfer between ZnSe and Eu. The intrinsic mechanism of energy transfer with different ZnS shell thickness was systematically investigated through time-resolved spectra and energy transfer dynamics theory. The results revealed that energy was transmitted in the form of dipole-electric dipole interaction.

## Abbreviations

*I*_613_: The PL intensity integral of the Eu ion
*I*_*B*_: The band edge PL intensity integral of ZnSe
*I*_*D*_: The defect-related luminescence intensity integral of ZnSe
PL: Photoluminescence
QDs: Quantum dots
TEM: Transmission electron microscopy
XRD: X-ray diffraction
